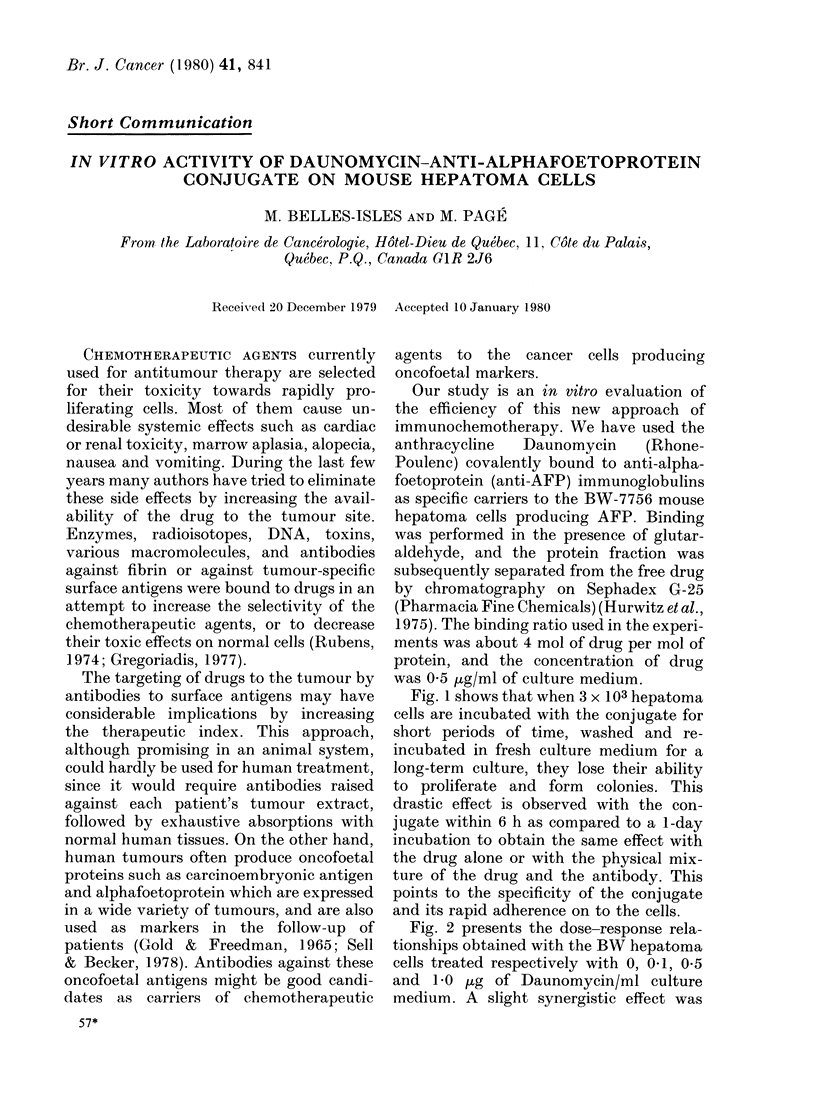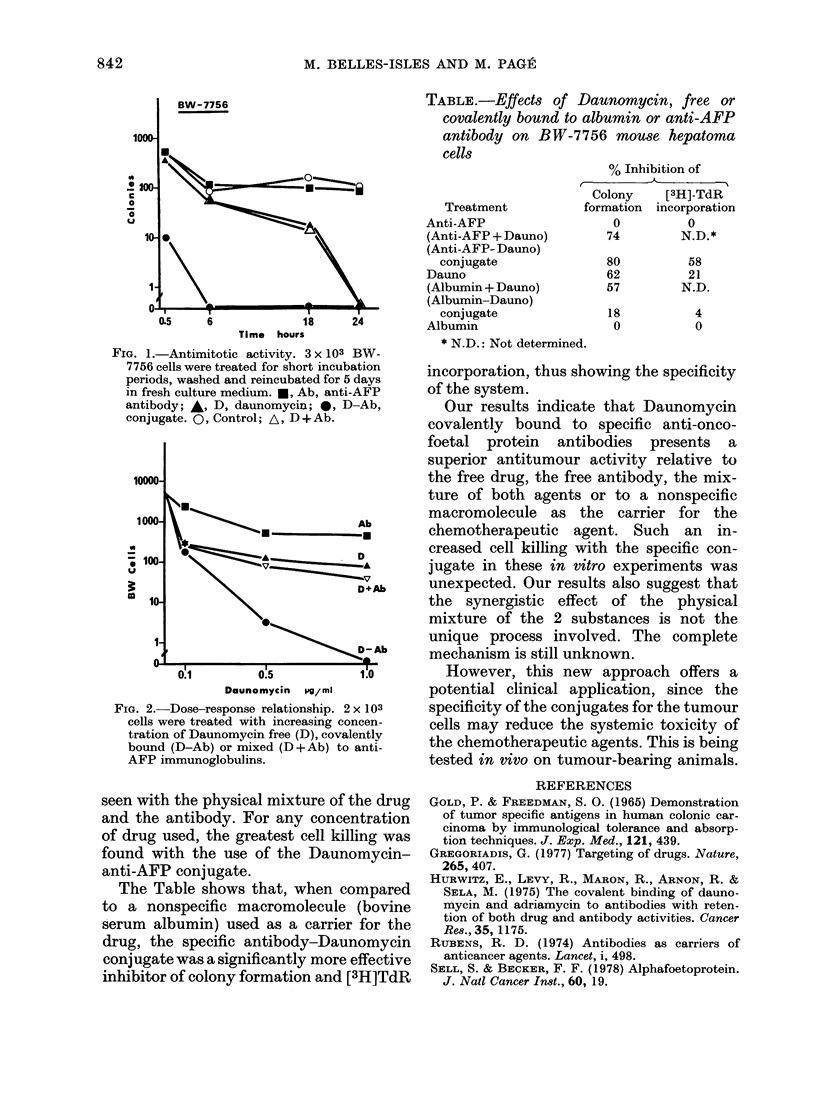# In vitro activity of daunomycin-anti-alphafoetoprotein conjugate on mouse hepatoma cells.

**DOI:** 10.1038/bjc.1980.153

**Published:** 1980-05

**Authors:** M. Belles-Isles, M. Pagé


*Br. J. Cancer* (1980) **41,** 841

***Short Communication***

### *IN VITRO* ACTIVITY OF DAUNOMYCIN–ANTI-ALPHAFOETOPROTEIN CONJUGATE ON MOUSE HEPATOMA CELLS

M. BELLES-ISLES AND M. PAGÉ

*From the Laboratoire de Cancérologie, Hôtel-Dieu de Québec,* 11, *Côte du Palais, Québec, P.Q., Canada G1R 2J6*

Received 20 December 1979 Accepted 10 January 1980

CHEMOTHERAPEUTIC AGENTS currently used for antitumour therapy are selected for their toxicity towards rapidly proliferating cells. Most of them cause undesirable systemic effects such as cardiac or renal toxicity, marrow aplasia, alopecia, nausea and vomiting. During the last few years many authors have tried to eliminate these side effects by increasing the availability of the drug to the tumour site. Enzymes, radioisotopes, DNA, toxins, various macromolecules, and antibodies against fibrin or against tumour-specific surface antigens were bound to drugs in an attempt to increase the selectivity of the chemotherapeutic agents, or to decrease their toxic effects on normal cells (Rubens, 1974; Gregoriadis, 1977).

The targeting of drugs to the tumour by antibodies to surface antigens may have considerable implications by increasing the therapeutic index. This approach, although promising in an animal system, could hardly be used for human treatment, since it would require antibodies raised against each patient's tumour extract, followed by exhaustive absorptions with normal human tissues. On the other hand, human tumours often produce oncofoetal proteins such as carcinoembryonic antigen and alphafoetoprotein which are expressed in a wide variety of tumours, and are also used as markers in the follow-up of patients (Gold & Freedman, 1965; Sell & Becker, 1978). Antibodies against these oncofoetal antigens might be good candidates as carriers of chemotherapeutic agents to the cancer cells producing oncofoetal markers.

Our study is an *in vitro* evaluation of the efficiency of this new approach of immunochemotherapy. We have used the anthracycline Daunomycin (Rhone-Poulenc) covalently bound to anti-alphafoetoprotein (anti-AFP) immunoglobulins as specific carriers to the BW-7756 mouse hepatoma cells producing AFP. Binding was performed in the presence of glutaraldehyde, and the protein fraction was subsequently separated from the free drug by chromatography on Sephadex G-25 (Pharmacia Fine Chemicals) (Hurwitz *et al.*, 1975). The binding ratio used in the experiments was about 4 mol of drug per mol of protein, and the concentration of drug was 0·5 $\mu$g/ml of culture medium.

Fig. 1 shows that when $3 \times 10^3$ hepatoma cells are incubated with the conjugate for short periods of time, washed and reincubated in fresh culture medium for a long-term culture, they lose their ability to proliferate and form colonies. This drastic effect is observed with the conjugate within 6 h as compared to a 1-day incubation to obtain the same effect with the drug alone or with the physical mixture of the drug and the antibody. This points to the specificity of the conjugate and its rapid adherence on to the cells.

Fig. 2 presents the dose–response relationships obtained with the BW hepatoma cells treated respectively with 0, 0·1, 0·5 and 1·0 $\mu$g of Daunomycin/ml culture medium. A slight synergistic effect was

FIG. 1.—Antimitotic activity. $3 \times 10^3$ BW-7756 cells were treated for short incubation periods, washed and reincubated for 5 days in fresh culture medium. ■, Ab, anti-AFP antibody; ▲, D, daunomycin; ●, D–Ab, conjugate. ○, Control; △, D+Ab.

FIG. 2.—Dose-response relationship. $2 \times 10^3$ cells were treated with increasing concentration of Daunomycin free (D), covalently bound (D–Ab) or mixed (D+Ab) to anti-AFP immunoglobulins.

seen with the physical mixture of the drug and the antibody. For any concentration of drug used, the greatest cell killing was found with the use of the Daunomycin–anti-AFP conjugate.

The Table shows that, when compared to a nonspecific macromolecule (bovine serum albumin) used as a carrier for the drug, the specific antibody–Daunomycin conjugate was a significantly more effective inhibitor of colony formation and [$^3$H]TdR

TABLE.—*Effects of Daunomycin, free or covalently bound to albumin or anti-AFP antibody on BW-7756 mouse hepatoma cells*

| | % Inhibition of | |
|---|---|---|
| Treatment | Colony formation | [$^3$H]-TdR incorporation |
| Anti-AFP | 0 | 0 |
| (Anti-AFP + Dauno) | 74 | N.D.* |
| (Anti-AFP-Dauno) conjugate | 80 | 58 |
| Dauno | 62 | 21 |
| (Albumin + Dauno) | 57 | N.D. |
| (Albumin–Dauno) conjugate | 18 | 4 |
| Albumin | 0 | 0 |

* N.D.: Not determined.

incorporation, thus showing the specificity of the system.

Our results indicate that Daunomycin covalently bound to specific anti-oncofoetal protein antibodies presents a superior antitumour activity relative to the free drug, the free antibody, the mixture of both agents or to a nonspecific macromolecule as the carrier for the chemotherapeutic agent. Such an increased cell killing with the specific conjugate in these *in vitro* experiments was unexpected. Our results also suggest that the synergistic effect of the physical mixture of the 2 substances is not the unique process involved. The complete mechanism is still unknown.

However, this new approach offers a potential clinical application, since the specificity of the conjugates for the tumour cells may reduce the systemic toxicity of the chemotherapeutic agents. This is being tested *in vivo* on tumour-bearing animals.

### REFERENCES

GOLD, P. & FREEDMAN, S. O. (1965) Demonstration of tumor specific antigens in human colonic carcinoma by immunological tolerance and absorption techniques. *J. Exp. Med.*, **121**, 439.

GREGORIADIS, G. (1977) Targeting of drugs. *Nature*, **265**, 407.

HURWITZ, E., LEVY, R., MARON, R., ARNON, R. & SELA, M. (1975) The covalent binding of daunomycin and adriamycin to antibodies with retention of both drug and antibody activities. *Cancer Res.*, **35**, 1175.

RUBENS, R. D. (1974) Antibodies as carriers of anticancer agents. *Lancet*, i, 498.

SELL, S. & BECKER, F. F. (1978) Alphafoetoprotein. *J. Natl Cancer Inst.*, **60**, 19.